# Therapeutic potential of archaeal unfoldase PAN^et^ and the gateless T20S proteasome in P23H rhodopsin retinitis pigmentosa mice

**DOI:** 10.1371/journal.pone.0308058

**Published:** 2024-10-03

**Authors:** Celine Brooks, Douglas Kolson, Emily Sechrest, Janelle Chuah, Jane Schupp, Neil Billington, Wen-Tao Deng, David Smith, Maxim Sokolov

**Affiliations:** 1 Department of Ophthalmology, West Virginia University, Morgantown, West Virginia, United States of America; 2 Department of Biochemistry and Molecular Medicine, West Virginia University, Morgantown, West Virginia, United States of America; 3 Department of Neuroscience, West Virginia University, Morgantown, West Virginia, United States of America; Carl von Ossietzky Universitat Oldenburg, GERMANY

## Abstract

Neurodegenerative diseases are characterized by the presence of misfolded and aggregated proteins which are thought to contribute to the development of the disease. In one form of inherited blinding disease, retinitis pigmentosa, a P23H mutation in the light-sensing receptor, rhodopsin causes rhodopsin misfolding resulting in complete vision loss. We investigated whether a xenogeneic protein-unfolding ATPase (unfoldase) from thermophilic *Archaea*, termed PAN^et^, could counteract the proteotoxicity of P23H rhodopsin. We found that PAN^et^ increased the number of surviving photoreceptors in P23H rhodopsin mice and recognized rhodopsin as a substate *in vitro*. This data supports the feasibility and efficacy of using a xenogeneic unfoldase as a therapeutic approach in mouse models of human neurodegenerative diseases. We also showed that an archaeal proteasome, called the T20S can degrade rhodopsin *in vitro* and demonstrated that it is feasible and safe to express gateless T20S proteasomes *in vivo* in mouse rod photoreceptors. Expression of archaeal proteasomes may be an effective therapeutic approach to stimulate protein degradation in retinopathies and neurodegenerative diseases with protein-misfolding etiology.

## Introduction

Misfolded and damaged proteins are a hallmark of aging and many neurodegenerative diseases [1, 2] including diseases causing blindness [3, 4]. Retinitis pigmentosa (RP) is one such blinding disease characterized by a gradual loss of retinal photoreceptors resulting in progressive vision loss and ultimately, blindness with a prevalence of one in 4000 people [5, 6]. Currently, there is no cure for this disease and there are few effective treatments. RP has been linked to hundreds of mutations in 71 different genes with autosomal dominant, autosomal recessive, or X-linked inheritance [7]. Mutations in the rhodopsin (RHO) gene, which encodes the G protein-coupled receptor responsible for initiating rod phototransduction, account for 25% of autosomal-dominant RP [8, 9].

The most prevalent rhodopsin mutation in the USA results in a proline-to-histidine substitution at position 23 (P23H), and it is responsible for 10% of all autosomal-dominant RP cases [10]. The P23H substitution, along with a number of other rhodopsin mutations implicated in RP, are thought to promote the misfolding of rhodopsin [11, 12]. Knock-in P23H rhodopsin mice phenotypically recapitulate an aggressive form of human RP demonstrated by the complete loss of their rods and cones by six months of age [13]. The amount of rhodopsin in these mice is reduced, as virtually all P23H rhodopsin is degraded by the proteasome [14] and lysosome [15]. However, this phenotype does not appear to be caused by haploinsufficiency, because mice lacking one copy of the opsin gene experience minimal photoreceptor loss [16]. P23H rhodopsin is proposed to stimulate apoptosis by either triggering ER stress and activating the unfolded protein response (UPR) [17, 18] or disrupting the organization of rod outer segments [13, 19], however the precise mechanism behind P23H rhodopsin cytotoxicity is unknown.

We along with others [20, 21] have been targeting the proteasome system to promote protein degradation and preserve vision in P23H rhodopsin mice. P23H rhodopsin is proposed to be degraded by the proteasome through ER-associated protein degradation (ERAD) [22]. However, rhodopsin is a highly abundant protein with 60 million rhodopsin molecules per photoreceptor [23], and up to ten million rhodopsin molecules are synthesized each day [24]. As a result of its high concentration, P23H rhodopsin may place a significant strain on the proteasome indeed, P23H rhodopsin mice were shown to have reduced proteasome capacity [25] and increasing proteasome levels by overexpressing transcription factor, nuclear factor erythroid-2-like 1 (Nfe2l1) improved vision in P23H rhodopsin mice [21]. The majority of protein degradation is carried out by the 26S proteasome [26], which is made up of the 19S regulatory particle and 20S core. The 20S core consists of four stacked heteroheptameric rings in an α-β-β-α arrangement. The N-termini of the α-subunits form a physical gate above the proteolytic β-subunits to prevent unregulated protein degradation. Gate opening is stimulated by the ATPase subunits of the 19S which are also responsible for unfolding and inserting substrates into the 20S chamber for degradation. Truncation of α’s N-terminus creates a gateless or constitutively open 20S and expression of gateless 20S has been shown to reduce the amount of proteotoxic proteins, tau and α-synuclein in HEK 293 cells [27].

Our strategy to stimulate protein turnover was to express the archaeal proteasome. The archaeal proteasome contains an ATPase complex called PAN and a 20S core. Similar to the ATPase subunits of the 19S, PAN is responsible for unfolding and translocating clients into the 20S core, where they are cleaved into short peptides. Previously, we described functional expression of epitope-tagged PAN^et^ in rod photoreceptors of transgenic mice [28]. The C-terminal epitope-tag containing HA and FLAG prevents physical association of PAN^et^ with the T20S, and most likely with the eukaryotic 20S [29], thus converting this complex into a stand-alone unfoldase. Acting as an uncoupled unfoldase, PAN^et^ counteracted retinal degeneration in a generic model of protein misfolding retinopathy, Gγ_1_ knockout mice. The present study aims to expand these findings by testing the efficacy of PAN^et^ in P23H rhodopsin mice, a more clinically relevant mouse model. Our rationale was that PAN^et^ will alleviate the cytotoxicity of misfolded proteins, including P23H rhodopsin, by promoting their unfolding and degradation by the endogenous proteasome. In addition, we assessed the feasibility and safety of expressing gateless 20S from thermophilic archaea, Thermoplasma acidophilum (T20S) in rod photoreceptors of wild-type and PAN^et^ mice. This was the first study to express an archaeal proteasome in a model organism and provide proof of concept for future studies to determine the efficacy of archaeal proteasomes in models of neurodegenerative diseases.

## Materials and methods

### Animal models

The use of animal models in this study conforms to ARVO’s Statement for the Use of Animals in Ophthalmic and Vision Research, and this study was approved by the West Virginia University Institutional Animal Care and Use Committee (protocol 1603001702). PAN^et^ mice [28], Rho^P23H/WT^ mice [13], and Ub^G76V^-GFP mice [30] were back-crossed into a wild-type genetic background (129-E, Charles River) for at least ten generations. The resulting isogenic strains were crossed with each other, and their offspring was ear-tagged and genotyped using PCR amplification of genomic DNA as described in the original papers. Mice were housed in adjacent cages, separated by gender but not by genotype, under standard diurnal cycle. At the defined ages, visual responses of retinal photoreceptors were analyzed by ERG. Then, the mice were euthanized, using CO_2_ inhalation followed by cervical dislocation, and their eyes collected and sent to Excalibur Pathology, Inc (Norman, OK, USA) for histology processing.

### Electroretinography (ERG)

Mice were dark-adapted overnight prior to testing, and all procedures were performed under dim red light. Mice were anesthetized by 1.5% isoflurane with 2.5 liters per minute (lpm) oxygen delivered through a nose cone. The animals’ pupils were dilated with a mixture of 1.25% phenylephrine hydrochloride and 0.5% tropicamide ophthalmic solution. Visual responses were recorded simultaneously from both eyes using either a Celeris (Diagnosis LLC) or an UTAS BigShot (LKC Technologies) rodent ERG system.

### Automated photoreceptor nuclei counting

Total nuclei count within the outer nuclear layer (ONL) was determined in continuous ocular cross-sections stained with hematoxylin and eosin. The specimens were digitized using an Olympus VS120 slide scanner. The resulting TIFF images, with 0.344 μm pixel size, were analyzed in the red, blue and green channels using a custom Cell Profiler pipeline named ONLyzer.cpproj (available as a supplementary download), as outlined below. A description of the function of each step is included in the annotated pipeline.

The retinal pigmented epithelium (RPE) is first identified in the red channel and used as a positional reference. Nuclei are then detected as objects in the expected size range, 4–20 pixels, using a source image derived from the product of the blue and green channels divided by the red channel. Outputs include a version of the original image overlaid with detected nuclei. Each output is inspected for spurious detections and, if needed, subjected to additional refinement. Most commonly, this included applying several intensity- and area-based filters, while using the RPE to mask the dataset. In cases where selected nuclei extended into the inner nuclear layer, the pipeline was run using a more stringent filter based on the distance from the RPE. The position of this filter was chosen by looking at the histogram of the first output and choosing a cutoff which would include only the first peak of the bimodal distribution (see Supplementary info for an example of this). At the advanced retinal degeneration states, some detections of ONL nuclei underperformed due to the pipeline requirements for large groups of nuclei to be detected in clusters. These datasets were therefore re-run with a more sensitive threshold for initial detection, a lower threshold of group size and a stringent filter to remove any detections beyond a specified value from the RPE. Measurements of total nuclei detected per image were output as Excel files.

### AAV-T20S-αΔN design and production

The coding sequence of T20S-αΔN was codon optimized for mammalian expression synthesized by Genscript and inserted into pcDNA3.1+. T20S-αΔN consisted of the cDNA sequence from Thermoplasma acidophilum encoding the proteasome β-subunit (PsmB) followed by the proteasome α-subunit (^Δ2-11^PsmA) with an internal ribosome entry site (IRES) between the β and α subunits. DNA bases corresponding to amino acids 2–11 of the proteasome α-subunit were removed (^Δ2-11^PsmA) to create gateless proteasome, T20S-αΔN. A 6X-His tag was added to the C-terminus of the β-subunit to enable detection and purification. Initially, the AAV plasmid [31] carried humanized green fluorescent protein (hGFP) under the mouse opsin promoter (mOP) which limits the expression of hGFP to rod photoreceptors. T20S-αΔN was moved to the AAV plasmid by replacing GFP with the coding sequence of T20S-αΔN. mOP-T20S-αΔN was packaged into AAV-PHP.eB capsid [32]. AAV was produced by triple-plasmid transfection of HEK293T cells cultured in 5-layer tissue culture flasks (870 cm^2^). At about 70% confluency, cells were transfected with pHGTIadeno1 (adenovirus helper plasmid; John T. Gray and Harvard College), pUCmini-iCAP-PHP.eB (AAV9 derived helper plasmid) and AAV-mOP-T20S-αΔN in complex with PEI. Three days post transfection, cells and culture medium were collected and any AAV present in the clarified medium was precipitated with 0.25 medium volume of 40% PEG800/2.5 M NaCl at 4⁰C overnight followed by centrifugation at 2000xg for 1 hour [33]. Then, the medium precipitate was resuspended in 2X PBS. The cell pellet was resuspended in lysis buffer (50 mM TRIS/HCl, 50 mM NaCl, 10 mM MgCl_2_, 500 U of DNA nuclease, pH 8.0), supplemented with 2% Triton X-100 and incubated 20 minutes at 37⁰C. Then, the lysate was supplemented with 1% sodium deoxycholate (10% in water) and incubated for an additional 10 minutes at 37⁰C. Lastly, the lysate and the resuspended medium precipitate were combined, supplemented with 0.4M NaCl and clarified by centrifugation (30 min at 7000xg). The clear lysate was purified by chromatography using a 1 mL AVIPure®-AAV9 column from Repligen (Waltham, MA, USA) as per the manufacturer’s protocol. The eluted virus was concentrated, dialyzed against PBS, supplemented with 0.02% Pluronic F-68 and filter-sterilized. Viral titer was estimated by the alkaline agarose gel method [34].

### Tissue culture

HEK 293 cells were maintained in DMEM/F-12 medium supplemented with 10% FBS and 1% penicillin-streptomycin in an incubator at 37⁰C and 5% CO_2_. For transient T20S-αΔN expression, cells were plated in six-well plates and grown to 80% confluency. The following day, cells were transfected with 2.5 μg of plasmid DNA using polyethylenimine (PEI) at a 1 (plasmid DNA): 2.5 (PEI) ratio. Empty pcDNA3.1+ vector was used as a control. Cells were collected 48h after transfection, frozen on dry ice, and stored at -80⁰C.

### Subretinal AAV injections

Eyes of 1-2-month-old mice were dilated 15–30 minutes prior to anesthesia and subretinal injection using Tropi-Phen drops (Pine Pharmaceuticals, Tonawanda, NY). Mice were anesthetized by intramuscular injection of ketamine (80 mg/kg) and xylazine (10 mg/kg) in sterile phosphate buffered saline (PBS). A drop of GenTeal was placed on the cornea to make the inside of the injected eye clearly visible under microscope. Next, an entering puncture was placed with a 25-gauge needle at the edge of the cornea and then trans-corneal subretinal injections were completed using a 33-gauge blunt end needle attached to a 5 μl Hamilton syringe. AAV mixed with fluorescein dye (0.1% final concentration, 1 μl volume of mixture) was delivered into the subretinal space as described previously [35, 36]. Following injection, eyes were treated with Neomycin/Polymixin B Sulfates/Bacitracin Zinc ophthalmic ointment (Bausch & Lomb, Inc., Tampa, FL). To reverse anesthesia, mice were administered an intraperitoneal injection of antisedan (Orion Corporation, Espoo, Finland), and the animal was kept warm until recovered from anesthesia.

### Pulldown of T20S-αΔN

HEK293 cells from one well of a six well plate or 8–10 mouse retinas were homogenized in 0.4mL of 20mM Tris/HCl, pH 7.5, 100mM NaCl, and 1.0% Triton X-100 by short ultrasonic pulses. Resulting cell/tissue extracts were cleared by centrifugation. T20S-αΔN was captured with 5μL of HisPur Ni-NTA resin (88221, Thermo Fisher) and eluted with 300mM imidazole. Alternatively, T20S-αΔN was immunoprecipitated with 1μg of 6x-His tag mouse monoclonal antibody and 10μL of protein A/G agarose (20421, Thermo Fisher). Captured proteins were eluted with 3% ammonia solution, and vacuum-dried. HEK 293 cells transfected with empty pcDNA3.1+ vector was used as a control for Ni-NTA and 6X His pulldowns.

### Quantification of the Ub^G76V^-GFP reporter in the retina

Retinas were collected at one month of age. Four retinas were homogenized in 0.4 mL of RIPA buffer (R0278, Sigma) by short ultrasonic pulses. Resulting retinal extracts were cleared by centrifugation; the protein concentration in the extracts was determined by the BCA assay (23225, Pierce, Thermo Fisher Scientific). The extract was incubated with 10μL of anti-GFP magnetic beads (gtma, Chromotek) for 1 hour at room temperature. Beads were washed four times for 3 minutes with RIPA buffer. The captured proteins were eluted with urea sample buffer (USB; 125mM Tris/HCl, pH 6.8, 4% SDS, 6M Urea and 2.5% β-mercaptoethanol) for 10 minutes at 95⁰C. Equal aliquots of the eluates were separated on a Novex WedgeWell 10%–20% Tris-Glycine Gel (XP10202BOX, Invitrogen, Thermo Fisher Scientific), transferred to Immobilon FL membrane (IPFL00010, Millipore), analyzed by Western blotting, and imaged using an Odyssey Infrared Imaging System (LI-COR Biosciences) according to the manufacturer’s protocol. The GFP signals were normalized to the total protein concentration in the retinal extracts.

*Quantification*: To reduce variability, anti-GFP pulldowns from PAN^et^(-) and PAN^et^(+) groups were conducted simultaneously and separated in adjacent wells for Western blotting. GFP signals were normalized to total protein concentration in the retinal extracts that were used for pulldown. Then, the adjusted value in PAN^et^(+) sample was expressed as percentage of that in PAN^et^(-) sample. The experiment was repeated 6 times and the mean difference PAN^et^(-) and PAN^et^(+) groups was calculated. Significance was determined by paired t-test.

### Measuring transcript levels for rhodopsin and Ub^G76V^-GFP

Retinas were dissected and frozen on dry ice. RNA was isolated using the RNeasy Mini Kit (74104, Qiagen) and reverse transcribed into cDNA using the Protoscript II Kit (E6560S, New England Biolabs). Samples were analyzed by qPCR using Brilliant II SYBR Green qPCR Master Mix (600828, Stratagene) on the Mx3000P qPCR System (Agilent/Stratagene) or the QuantStudio 6 Flex Real-Time PCR System (Applied Biosystems). The relative transcript levels for the genes of interest were measured using the following primers: Rho-F (GAA TCA CGC TAT CAT GGG TGT GG) and Rho-R (ATG ACA AAG GAT TCG TTG TTG ACC) for wild-type and P23H rhodopsin (Rho); GFP-F (ACA GCC ACA ACG TCT ATA TCA TGG) and GFP-R (GTG TTC TGC TGG TAG TGG TCG) for UbGFP; and Ywhaz-F (GTT GTA GGA GCC CGT AGG TCA TCG) and Ywhaz-R (GCT TTC TGG TTG CGA AGC ATT GGG) for Tyrosine 3-Monooxygenase/Tryptophan 5-Monooxygenase Activation Protein Zeta (Ywhaz), used as a reference gene.

### Proteolytic degradation of rhodopsin by PAN-T20S

Purification of recombinant PAN and T20S was performed as described previously [37, 38]. GFP-ssrA, used as a surrogate substrate of PAN-T20S, was purified as in [39]. Rhodopsin was immunoprecipitated from one mouse retina with 4.0μg of 1D4 antibody (sc-57432, Santa Cruz Biotechnology) and 15.0μL of Pierce^TM^ Protein A/G UltraLink^TM^ Resin (53132, Thermo Fisher Scientific) in 0.2mL of T-PER^TM^ Tissue Protein Extraction Reagent (78510, Thermo Fisher Scientific). The captured rhodopsin was eluted with 0.1mL of 1% ammonia solution and vacuum-dried in 20.0μL aliquots. Prior to the assay, each rhodopsin aliquot was reconstituted in 0.1mL of the reaction buffer (30mM Tris/HCl pH 7.4, 5mM MgCl_2_, 10mM ATP, and 0.5% N-Dodecyl-β-Maltoside). The reaction was performed by mixing 10.0μL of rhodopsin solution with 0.5μL of PAN (1.0mg/mL) and 0.5μL of T20S (0.27mg/mL) and incubating for 3, 10, 20, 30, 40 minutes at 37⁰C. The reaction was stopped by adding 20.0μL of USB buffer, and the rhodopsin protein levels were assayed by Western blotting.

### Proteasome activity assay

To measure 20S proteasome activity, T20S-αΔN purified from HEK 293 cells was added to buffer containing 50 mM Tris-HCl (pH 7.4), 10mM MgCl_2_, 1mM DTT, and 100 mM suc-LLVY-amc. Hydrolysis of suc-LLVY-amc was monitored using a BioTek synergy 2 96-well plate reader at 45⁰C. Fluorescence was measured every 60 seconds for 1h (ex/em: 380/440 nm).

### Negative staining transmission electron microscopy (TEM)

T20S-αΔN purified from HEK 293 cells was negatively stained with 1% uranyl acetate on carbon film coated copper grids. TEM images were obtained using a JEOL 1010 Transmission Electron Microscope equipped with an AMT Hamamatsu ORCA-HR Digital Camera. 522 particles were aligned and classified into 24 classes using SPIDER software image processing. Of those 24 classes, the 9 clearest ones were selected to make the montage show in Fig 4E. The number of particles in the 9 selected classes is 268 (51% of the input particles). The full classification is shown in S1 Fig.

### Antibodies

Proteins were detected using the following antibodies: goat anti-GFP (Rockland, 600-301-215, IRDye800CW Conjugated), mouse anti-HA (Proteintech, 66006-2-Ig), mouse anti-ubiquitin (Invitrogen, eBioscience, 14-6078-82), mouse anti-rhodopsin (4D2), mouse anti-6x-His tag (MA1-21315, Invitrogen), and mouse anti-proteasome T20 α1,2,3,4,5,6 & 7 (MBL-PW8195-0100, Enzo).

### Statistical analyses

Real-time ERG data processing, including averaging of the repeated measurements, baseline drift compensation, and zero-line adjustment was carried out by the ERG software generating numerical values for the saturated a-wave at the end of the recording. Data in each group were analyzed combined, while treating right and left eyes independently, using SigmaPlot 13.0 (Systat Software, Inc). To generate the light-sensitivity curve, mean a-wave amplitude was calculated and plotted as a function of the stimulus flash intensity. The resulting plot was fitted with a single rectangular hyperbola with two parameters, $y=\frac{ax}{b+x}$. To determine significance, a-wave values in the two compared groups were analyzed by t-test generating P value. T-test was also used to determine the significance of Western blotting, RT-qPCR and ONL nuclei counting data. All graphs were generated in SigmaPlot 13.0.

## Results and discussion

### PAN^et^ increases rod photoreceptor survival in P23H opsin mice

**Fig 1:**
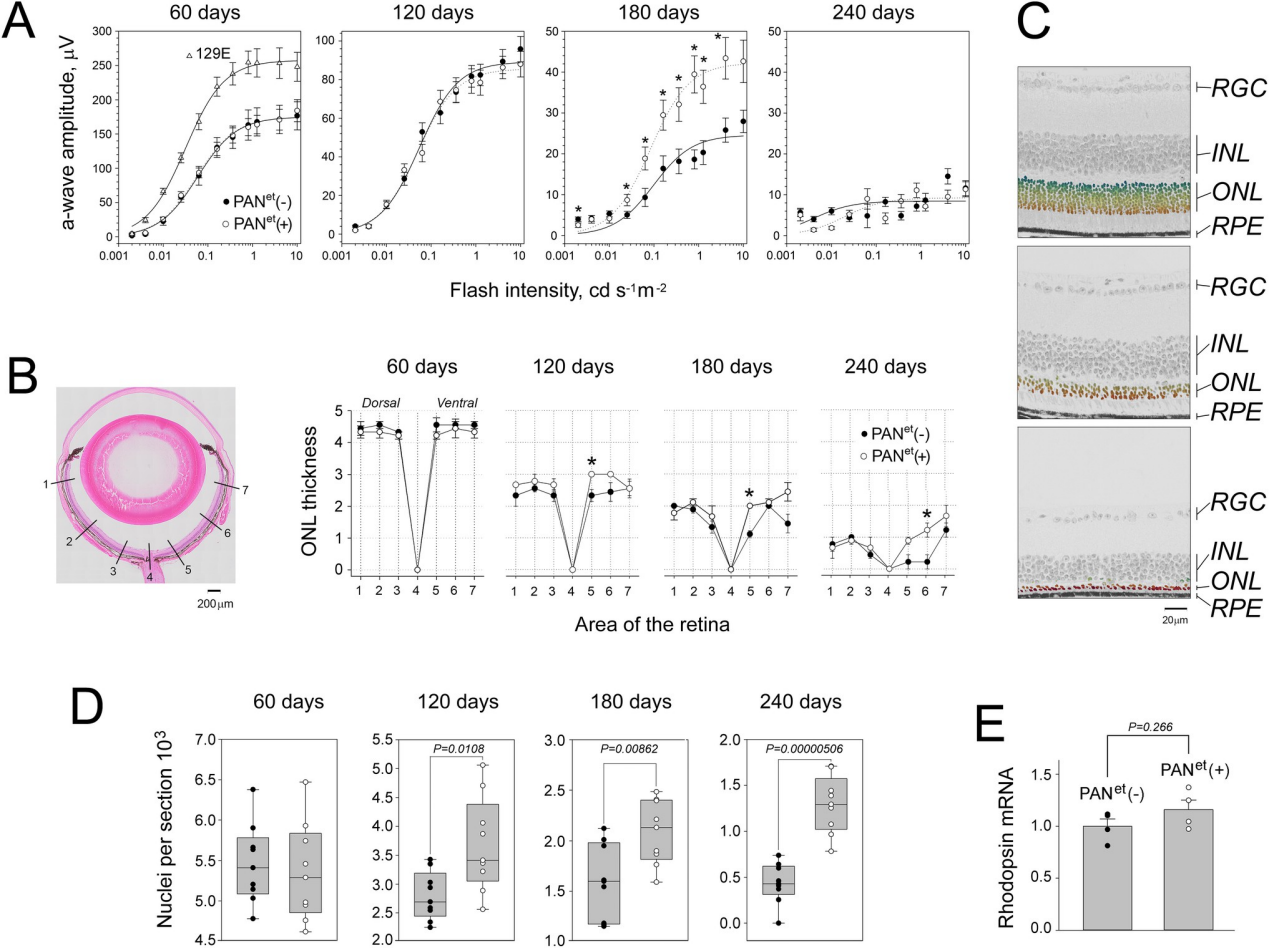
PAN^et^ increases rod survival in P23H rhodopsin mice. In all plots, PAN^et^ (-) Rho^P23H/WT^ (black circles) and PAN^et^ (+) Rho^P23H/WT^ (white circles) were compared at the indicated ages. Significance was determined using t-tests, showing two-tailed p-value or asterisk for P <0.05. ***A***, Visual responses of dark-adapted rods were recorded using a Celeris (Diagnosis LLC) rodent ERG system. The amplitude of elicited ERG a-wave is shown as a function of flash intensity (SEM, n = 8–10). Responses of wild type 129E mice at 60 days of age (open triangles) are shown for comparison. Each dataset was fitted with a simple rectangular hyperbola with two parameters using SigmaPlot 13 software. ***B***, *Panel*: Ocular cross-section stained with hematoxylin and eosin showing areas 1–7; *Graphs*: The number of stacked photoreceptors’ nuclei (ONL thickness) is plotted against the area number (SEM, n = 3). ***C***, Representative images of retinal cross-sections showing a portion of photoreceptor nuclei identified and color-coded by ONLyzer at different stages of retinal degeneration (top to bottom: age 30, 120 and 240 days); RGC − retinal ganglion cells, INL − inner nuclear layer, ONL − outer nuclear layer, RPE–retinal pigment epithelium. ***D***, Photoreceptor nuclei counts in the entire retinal cross-sections measured by ONLyzer. Morphologically intact cross-sections adjacent to the optic nerve, such as the one shown in B, were analyzed, generating the total nuclei count within the ONL. Distribution of numeric data with the 50% range, median value, and SD. E, Normalized levels of rhodopsin transcript determine by quantitative real-time PCR at 21 days of age (SEM, n = 4).

Mice carrying a P23H knock-in mutation of the rhodopsin gene (Rho) are widely utilized as a model for human RP caused by the P23H substitution, which is the most common rhodopsin mutation in the USA [8]. We crossed these P23H rhodopsin mice with PAN^et^ mice and compared the rate of rod photoreceptor death in PAN-positive and PAN-negative offspring carrying a single mutant rhodopsin allele, Rho^P23H/WT^. We used the amplitude of the dark-adapted electroretinography (ERG) a-wave as a readout for the number of surviving rods in the retina, since the a-wave is generated by the collective response of rods to light (Fig 1A). Between 60–120 days of age, PAN-positive and PAN-negative Rho^P23H/WT^ mice produced very similar a-waves. However, at 180 days of age PAN-positive mice produced significantly larger a-waves than their PAN-negative counterparts. Unexpectedly, this improvement was not maintained, and the a-waves were almost completely diminished by 240 days of age for both groups.

In addition to ERG, we monitored the progression of retinal degeneration by quantifying the retinal outer nuclear layer (ONL) which primarily consists of rod nuclei. The standard approach, based on manually counting the number of nuclei in a column at several designated areas (Fig 1B), was done in conjunction with an automated nuclei counting approach for retinal cross-sections (Fig 1C and 1D). Starting at 120 days of age, we found that PAN-positive mice had more surviving rods in the ventral retina, which degenerates faster than dorsal retina in Rho^P23H/WT^ mice [13] (Fig 1B). Consistent with this observation, PAN-positive mice maintained a higher rod count than PAN-negative mice in retinal cross-sections (Fig 1D). These data demonstrated a consistent protective effect of transgenic PAN^et^ in rod photoreceptors of P23H rhodopsin mice, which presented as a transient preservation of rod visual responses at 180 days of age.

To rule out that the PAN^et^ transgene interferes with rhodopsin transcription, which could decrease the biosynthesis of toxic P23H rhodopsin, we compared rhodopsin mRNA levels in the retinas of PAN^et^-negative and PAN^et^-positive Rho^P23H/WT^ mice by qPCR. We found that both groups showed similar rhodopsin transcript levels which indicates that the observed protective effect of PAN^et^ was not due to a reduction in gene expression (Fig 1E).

### PAN^et^ increases proteasomal load in rods expressing P23H rhodopsin

**Fig 2:**
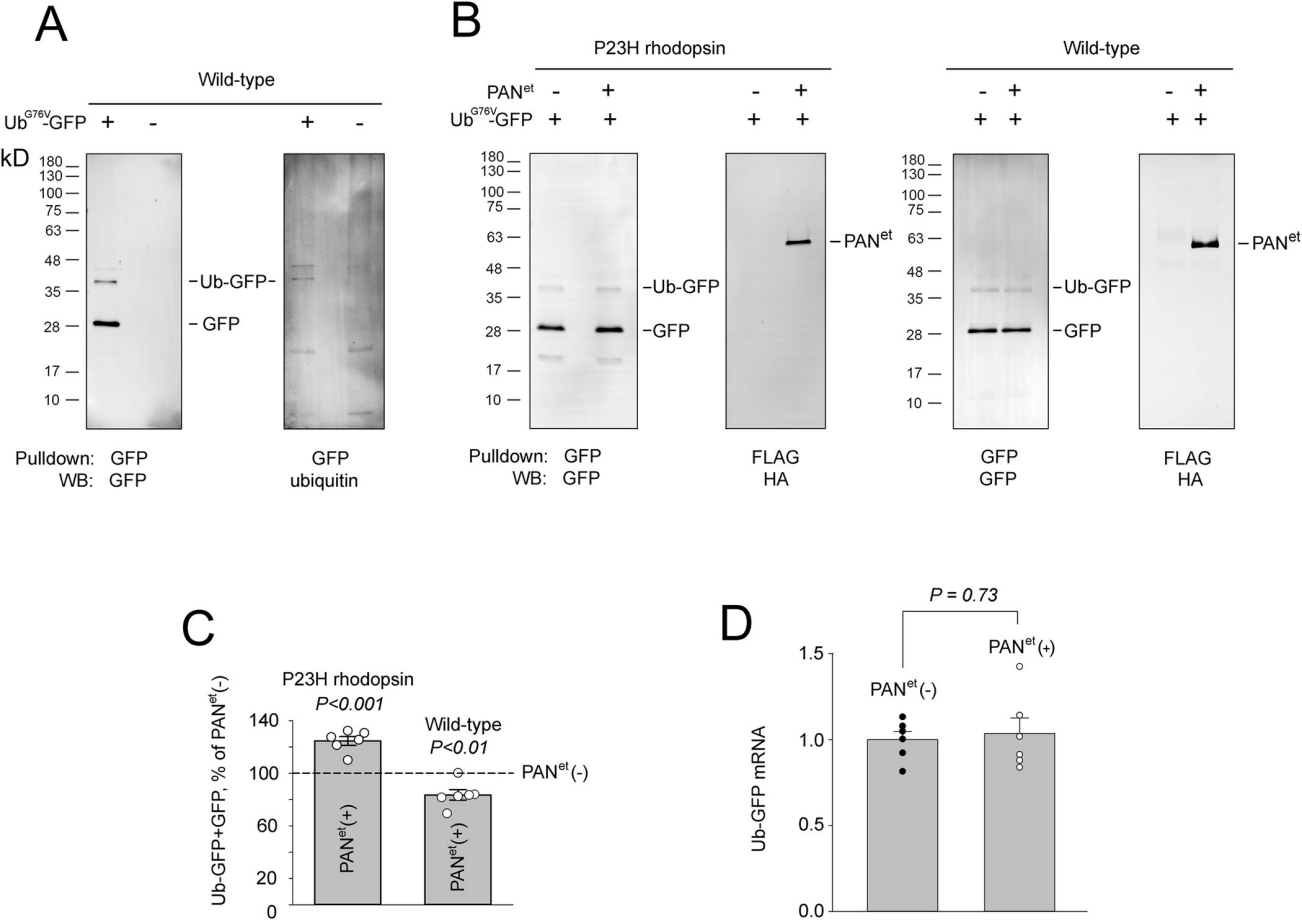
PAN^et^ increases proteasome load in P23H rhodopsin mice. ***A***, GFP pulldowns from retinas of wild-type mice expressing Ub^G76V^-GFP reporter and negative controls were analyzed by Western blotting. Bands corresponding to Ub-GFP and its GFP fragment are indicated. ***B***, Consecutive GFP and anti-FLAG pulldowns from the retinas of mice of the indicated genotype were analyzed by Western blotting. In all blots, 37kD and 28kD bands corresponding to Ub-GFP, and GFP fragment, respectively, are indicated. PAN^et^ containing FLAG and HA epitope tags runs as 55kD band. ***C***, Relative abundance of the combined Ub-GFP and GFP signals in retinas of PAN^et^(+) mice of P23H rhodopsin and wild-type backgrounds. Bar height shows mean percent value in PAN^et^(+) retinas compared to PAN^et^(-) retinas, with a combined Ub-GFP and GFP signal of 124.6±3.3% for P23H rhodopsin mice and 83.5±4.0% for wild-type mice (SEM, n = 6). Shown two-tailed p-values are from paired t-tests between PAN^et^(-) and PAN^et^(+) groups. ***D***, Normalized levels of Ub^G76V^-GFP transcript determined by quantitative real-time PCR (SEM, n = 6). In all plots, significance was determined using paired t-tests, showing two-tailed p-value or asterisk for P <0.05.

Next, we tested whether unfoldase PAN^et^ increased the amount of unfolded protein sent to the proteasome for degradation. The Ub^G76V^-GFP transgene has been used as proxy reporter substrate for the 26S proteasome [30, 40]. We used this reporter to evaluate the effect of PAN^et^ on the status of the 26S proteasome in rods of P23H rhodopsin mice. The Ub^G76V^-GFP transgene was introduced into PAN-positive and PAN-negative Rho^P23H/WT^ mice through cross-breeding. Previous work [25, 40] was able to detect Ub^G76V^-GFP in retinal lysates, however with our mouse background (129E) and GFP antibody, Ub^G76V^-GFP was barely detectable in lysates. Consequently, we quantified Ub^G76V^-GFP by capturing it from the retina with GFP-trap beads followed by Western blotting. The pulldown contained two bands, 28kD and 37kD in size that were recognized by antibodies against GFP (Fig 2A). Only the 37kD band was recognized by antibodies against ubiquitin. We concluded that the 37kD band was the full length Ub^G76V^-GFP reporter, whereas the 28kD band was a GFP fragment. A similar proteolytic cleavage of the reporter’s ubiquitin moiety was observed in cell culture [40]. The combined amount of reporter was 25±3% higher in PAN-positive compared to PAN-negative retinas (Fig 2B and 2C). The observed increase in the reporter is specific for rods expressing P23H rhodopsin because a slight but opposite effect of PAN was observed on wild-type background. There was no significant difference in the mRNA levels of Ub^G76V^-GFP between PAN-negative and PAN-positive Rho^P23H/WT^ mice (Fig 2D), which shows that the difference in protein levels was not due to transcriptional upregulation [41]. These data support the notion that PAN^et^ increases proteasomal load in rod photoreceptors of P23H rhodopsin mice.

### PAN-T20S recognizes and degrades rhodopsin

**Fig 3:**
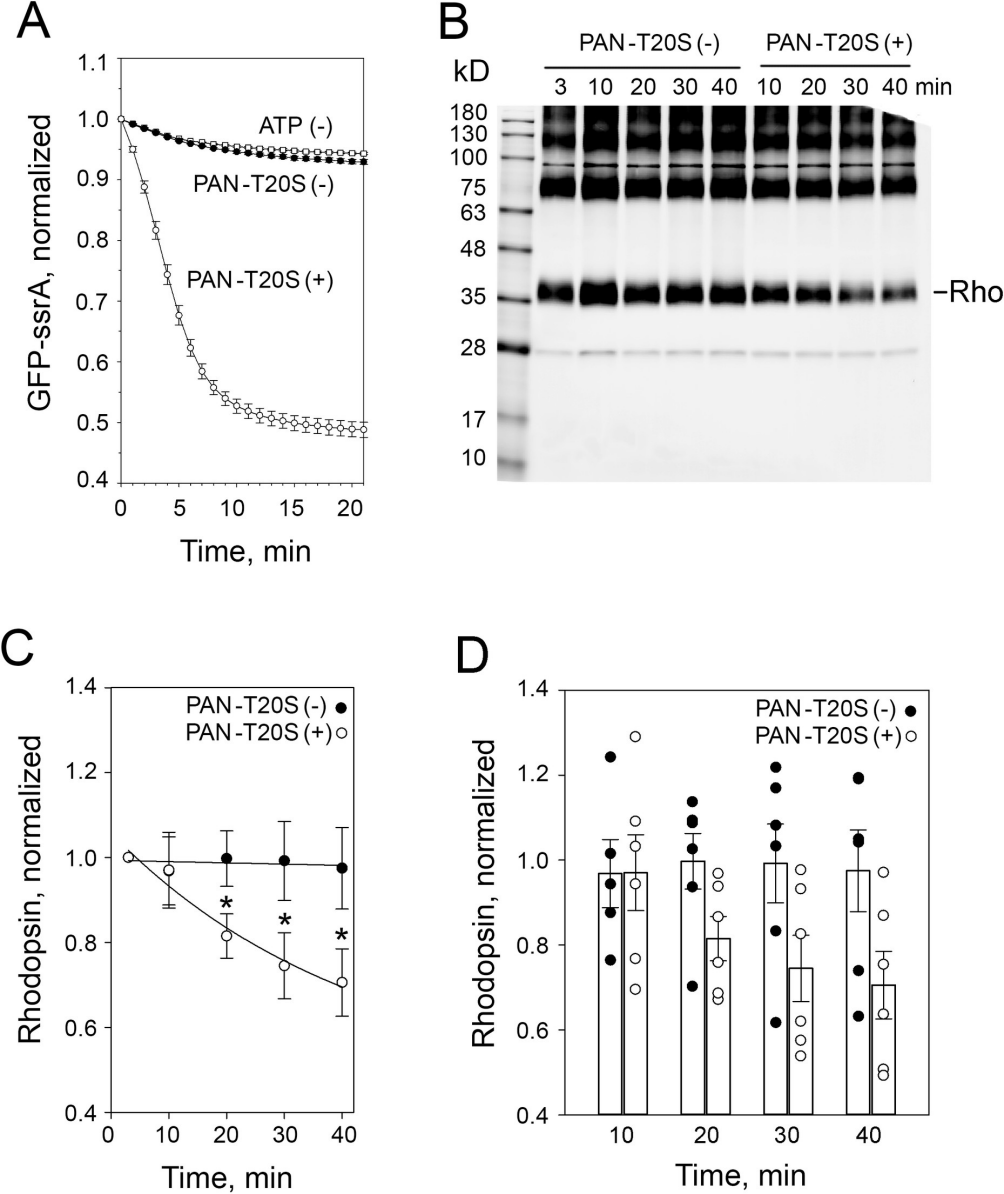
Proteolytic degradation of rhodopsin by recombinant archaeal PAN-T20S. ***A***, GFP-ssrA protein is degraded by recombinant PAN-T20S resulting in decreasing fluorescence over time. Data were normalized to control without PAN-T20S (see Materials and Methods for details). Error bars indicate SEM (n = 3). ***B***, Representative Western blotting showing degradation of rhodopsin isolated from mouse retina by PAN-T20S. ***C***, Time curve of rhodopsin degradation. Density of monomeric rhodopsin band (~35kD) at each time point was quantified. Each dataset was fitted with a single exponential decay function with three parameters using global curve fit. Error bars indicate SEM (n = 6). Data were compared using paired t-test. Asterisk indicates two-tailed p-value <0.05. ***D***, Spread of the data used in C with black and white circles representing normalized rhodopsin signal in the absence- and presence of PAN-T20S, respectively. Bar graphs show mean value with standard error at the indicated time points. Data at 3 min used for normalization is omitted from the plot.

Next, we tested whether PAN recognizes rhodopsin as a substrate and if an archaeal proteolytic core called the T20S could degrade this unfolded rhodopsin. First, we confirmed the activity of recombinant PAN-T20S using a well-established surrogate substrate, GFP-ssrA. We observed a real-time decrease of GFP fluorescence in this assay, which demonstrated that PAN-T20S unfolds and degrades GFP-ssrA (Fig 3A). Then, we presented PAN-T20S with rhodopsin isolated from mouse retina. The assay conditions were the same except rhodopsin was detected by Western blotting (Fig 3B). We observed a decrease in the intensity of the monomeric rhodopsin band over time in PAN-T20S samples compared to controls (Fig 3C and 3D). This result indicates that PAN recognizes rhodopsin as a substrate and rhodopsin is degraded by the T20S.

### Design of T20S-αΔN

**Fig 4:**
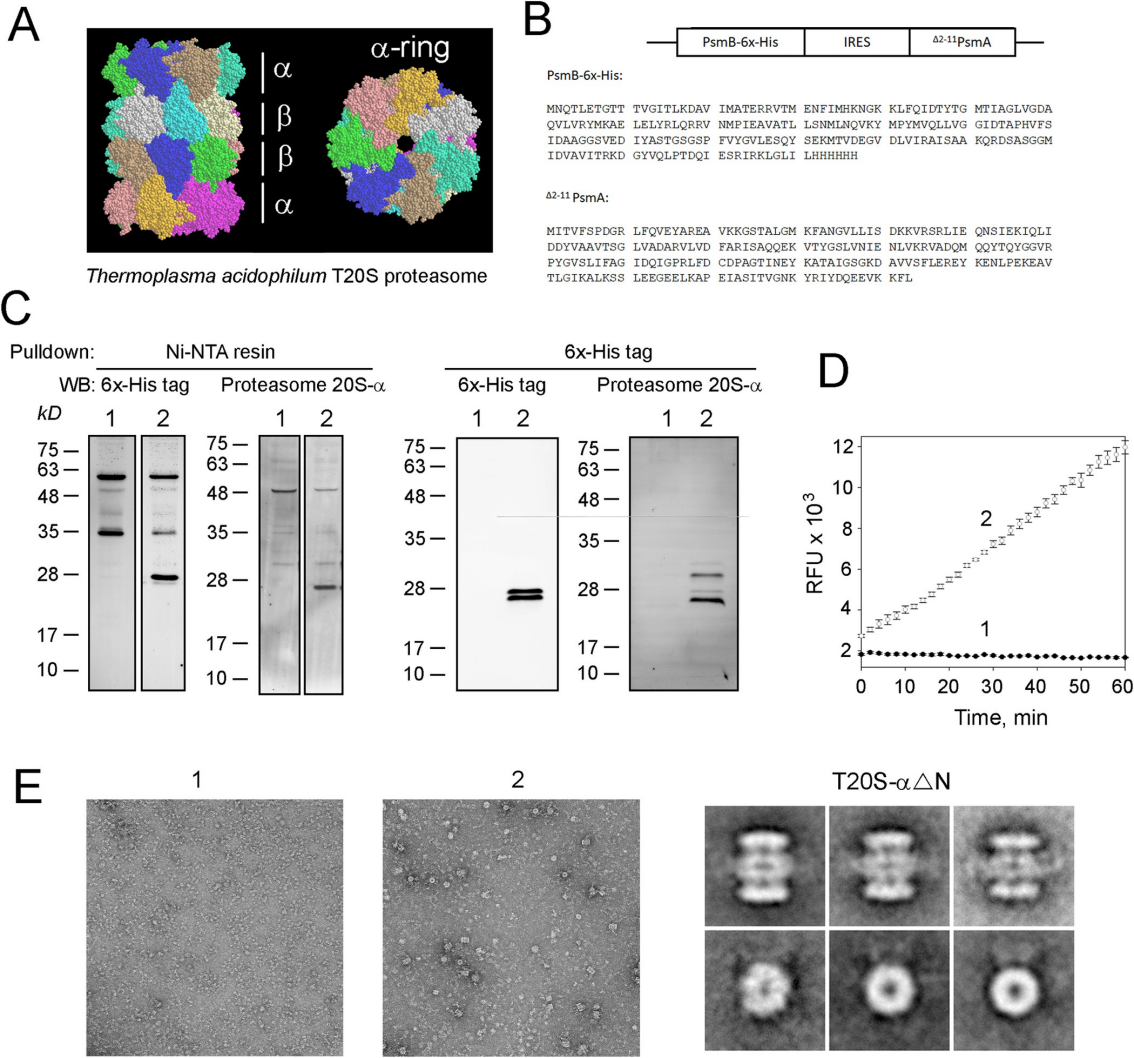
Expression of gateless archaeal 20S proteasome in HEK293 cells. ***A***, Side- and top-view of T20S proteasome from thermophilic archaea, Thermoplasma acidophilum comprised of two α-rings and two β-rings. Seven identical α or β subunits within each ring are shown in different colors (PDB ID 8F7K) ***B***, T20S-αΔN construct and the encoded sequences of the epitope-tagged β subunit (PsmB-6x-His) and the N-terminally truncated α subunit (^Δ2-11^PsmA). ***C***, Ni-NTA and anti-6x-His tag pulldowns from HEK293 cells, transfected with empty vector (1) or CMV-T20S-αΔN (2), were analyzed by Western blotting with indicated antibodies. ***D***, Chymotrypsin-like peptidase activity of T20S-αΔN, captured with Ni-NTA resin from HEK293 cells, transfected with empty vector (1) or CMV-T20S-αΔN (2), was determined with Suc-LLVY-AMC, a fluorogenic peptide substrate (SEM, n = 3). ***E***, Negative stain TEM of 6x-His tag antibody pulldowns from HEK293 cells transfected with empty vector (1) or CMV- T20S-αΔN (2) and global average side- and top-view of T20S-αΔN.

Given that PAN-T20S degraded rhodopsin, we wanted to test the feasibility and safety of expressing the T20S in mammalian cells and ultimately, rod photoreceptors. The T20S has homoheptameric α and β rings in an α-β-β-α arrangement similar to the eukaryotic 20S (Fig 4A). We designed the T20S with a 6X-His tag on the C-terminus of the β-subunit followed by an internal ribosome entry site (IRES) and the α-subunit with amino acids 2–11 removed (Fig 4B). These amino acids are known to form a physical gate over the β-subunits and their removal creates gateless or constitutively open T20S. We wanted to use gateless T20S, since the epitope tag on PAN^et^ prevents it from stimulating gate-opening and the T20S can degrade proteins by itself [42]. To be consistent with previous literature, we called this construct T20S-αΔN [43]. We purified T20S-αΔN from HEK293 cells and confirmed expression of the β and α subunits by Western blotting (Fig 4C). When T20S-αΔN was captured by Ni-NTA resin, it co-purified with several non-specific 35-60kD proteins that were absent when this complex was immunoprecipitated with antibody against anti-6X-His tag (Fig 4C). These non-specific proteins are likely endogenous mammalian proteins with poly-His sequences which may compete for Ni-NTA resin binding. The immunoprecipitated β-subunit appeared as two bands, likely representing proteolytically processed and unprocessed polypeptide; the β-subunit contains a N-terminal propeptide that is autocatalytically cleaved during complex assembly [44]. Overall, this confirms that mammalian cells can express an archaeal 20S proteasome, which was previously shown with 20S proteasomes from another archaeal species [45]. Next, we assessed T20S-αΔN’s ability to degrade a fluorogenic substrate. We found that T20S-αΔN isolated from HEK 293 cells displayed significant proteolytic activity (Fig 4D). To confirm that T20S-αΔN assembled into 20S proteasomes, we purified T20S-αΔN from HEK293 cells and performed TEM. We found that purified T20S-αΔN contained many particles with four stacked rings, which were absent from control pulldowns (Fig 4E). The blur around the β-rings is likely anti-His antibody which indicates that the 20S proteasomes come from T20S-αΔN and not endogenous mouse 20S proteasomes. Together these results indicate that archaeal T20S-αΔN can assemble into functionally active 20S proteasomes in mammalian cells. Given these encouraging results, we targeted the expression of T20S-αΔN to mouse rod photoreceptors using adeno-associated virus (AAV) carrying T20S-αΔN.

### Characterization of T20S-αΔN mice

**Fig 5:**
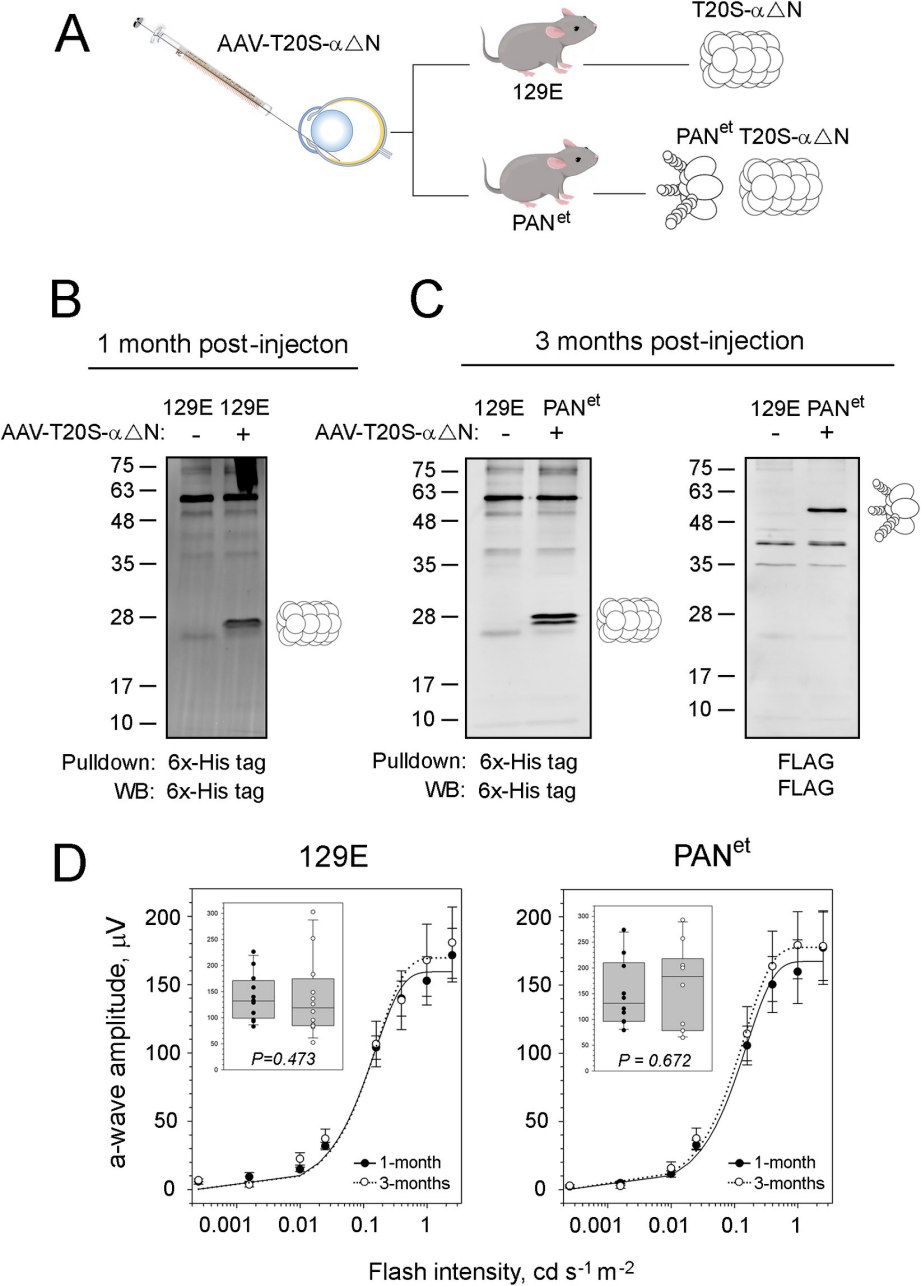
T20S-αΔN is nontoxic for rod photoreceptors. ***A***, Experimental design: 129E and PAN^et^ (+) mice were subretinally injected with AAV-T20S-αΔN vector, allowed to recover for 1- and 3 months, and analyzed by ERG and Western blotting. ***B***, T20S-αΔN detected by anti-6x-His pulldown and Western blotting in retinal extracts from noninjected and AAV-T20S-αΔN-injected 129E mice 1-month post-injection. ***C***, T20S-αΔN detected by anti-6x-His pulldown and Western blotting (left) and PAN^et^ detected by anti-FLAG pulldown and Western blotting (right) in retinal extracts from noninjected 129E mice and AAV-T20S-αΔN-injected PAN^et^ mice 3-month post-injection. ***D***, Comparison of visual responses one month and three months post-injection. Electroretinographic responses were recorded using a UTAS BigShot (LKC Technologies) rodent ERG system. The amplitude of elicited ERG a-wave is plotted as a function of flash intensity (SEM, n = 12). Each dataset was fitted with a simple rectangular hyperbola with two parameters. *Inset*: Distribution of numeric data with the 50% range, median value, and SD for the criterion flash of 0.4 cd s^-1^ m^-2^. The significance was determined by paired t-tests, showing two-tailed P value.

The cDNA of T20S-αΔN was cloned into an AAV vector under a rod-specific mouse opsin promoter (mOP) and packaged into AAV-PHP.eB capsid. AAV-T20S-αΔN was delivered to wild-type 129-E or PAN^et^ mice by subretinal injection into both eyes at one month of age (Fig 5A). Retinas were collected one and three months post-injection and T20S-αΔN and PAN expression were analyzed by Western blotting (Fig 5B). We confirmed expression of the T20S β-subunit in wild-type and PAN^et^ mice and expression of PAN in PAN^et^ mice. Additionally, expression of the T20S β-subunit and PAN persisted until at least 3 months post-injection (Fig 5B). We were unable to confirm expression of the T20S α-subunit due to low AAV expression of T20S-αΔN, which may have been exacerbated by an inefficient IRES-driven translation of the α-subunit. However, the presence of cleaved β-subunit indicates that the α-subunit is present because β’s assembly with α is required for its autocatalytic activity and removal of the propeptide [46]. To determine the impact of T20S-αΔN on rod function, we analyzed visual responses of AAV-T20S-αΔN injected wildtype and PAN^et^ mice one and three month post-injection by ERG. Visual responses were statistically indistinguishable across a wide range of stimulating flashes between one and three months post-injection for T20S-αΔN injected wildtype and PAN^et^ mice (Fig 5C). This indicates that expression of the gateless T20S proteasome (T20S-αΔN) does not reduce the viability or function of rod photoreceptors. Furthermore, expression of T20S-αΔN with PAN was also nontoxic to photoreceptors.

This study demonstrates that archaeal unfoldase, PAN^et^ recognizes rhodopsin as a substrate *in vitro* and provides a partial protective effect *in vivo* in P23H rhodopsin mice. At this point, we can only speculate how PAN^et^ increases the viability of rods in these mice. The observation that recombinant PAN-T20S degrades rhodopsin in our *in vitro* assay (Fig 3) suggests that PAN^et^ will likely recognize rhodopsin as substrate *in vivo*. Under basal conditions, rhodopsin is translated into the endoplasmic reticulum membrane and then promptly trafficked to the rod outer segment. Therefore, it is reasonable to assume that wild-type rhodopsin remains inaccessible to PAN^et^, which is a cytosolic complex excluded from the outer segment. Consistent with this, PAN^et^ had no obvious adverse effect on rhodopsin in wild-type mice [28]. However, during ERAD P23H rhodopsin is retro-translocated from the endoplasmic reticulum to the cytosol, where it can become accessible to PAN^et^ [45]. Subsequently, PAN^et^ can unfold P23H rhodopsin promoting its degradation by the proteasome. Some evidence also suggests that re-directing a large quantity of aggregation-prone rhodopsin to the cytosol increases the risk of aggregation and appearance of proteotoxic and degradation-resistant amyloids [14, 18, 47, 48]. By unfolding P23H rhodopsin, PAN^et^ may shift the balance from aggregation to degradation. This is supported by the observation that PAN^et^ increases the proteasomal load in rods expressing P23H rhodopsin, but not in wild type rods (Fig 2).

Unexpectedly, PAN^et^ did not start protecting rods in P23H rhodopsin mice until 120 days of age. P23H rhodopsin mice were shown to degenerate with biphasic kinetics with a rapid early loss of photoreceptors followed by a much slower phase [18]. Consistent with this, we found that 65% of photoreceptors die after 60 days of age, while the remaining 35% survive for an additional 180 days. It is possible that PAN^et^ can only protect photoreceptors during this slow phase of degeneration, which is consistent with our histological data. Although PAN^et^ increased the number of surviving rods in P23H rhodopsin mice between 120 and 240 days of age, PAN^et^ only increased rod visual responses at 180 days of age (Fig 1). Typically, rod outer segments which generate the ERG a-wave are lost before the photoreceptors dies. PAN^et^ may delay the breakdown of the outer segment, preserving ERG responses at 180 days of age. In our previous study, expression of PAN^et^ had a larger protective effect in Gγ_1_ knockout mice compared to P23H rhodopsin mice [28]. Gγ_1_ knockout mice [49] are thought to degenerate due to the misfolding of Gβ_1_ [40] which requires its binding partner, Gγ_1_ for folding. Recently, it was proposed that protein-unfolding contributes more to vision loss in Gγ_1_ knockout mice while protein degradation has a greater impact in P23H rhodopsin mice [50]. This may explain why PAN^et^, an unfoldase produced a larger rescue in Gγ_1_ knockout mice. It may also explain why overexpression of PA28α was more effective than PAN^et^ in P23H rhodopsin mice, since overexpression of PA28α increased proteasome activity in P23H rhodopsin mice [25]. Nevertheless, expression of PAN^et^ did increase photoreceptor survival in two models with protein-misfolding etiology, Gγ_1_ knockout and P23H rhodopsin mice. Future experiments will investigate the efficacy of PAN^et^ in additional mouse models of RP associated with rhodopsin misfolding such as the T17M mutation [51].

In this study, we also showed that expression of the gateless archaeal proteasome, T20S-αΔN from Thermoplasma acidophilum in mouse rod photoreceptors is feasible and nontoxic. A previous study showed functional expression of the archaeal Methanosarcina mazin 20S proteasome in mammalian cells using two vectors carrying either the α or β- subunit [45]. In our study, we coexpressed the α and β- subunits using an internal ribosome entry site (Fig 4B) and expression of both subunits (Fig 4C) confirms that our construct is functional. In mammalian cells, T20S-αΔN assembled into functionally active proteasomes (Fig 4D and 4E) suggesting that T20S-αΔN will assemble and retain its proteolytic activity *in vivo*. Expression of AAV-T20S-αΔN in mouse rod photoreceptors (Fig 5B) shows that a gateless archaeal proteasome can be expressed in mice. Additionally, there was no significant change in ERG a-waves of AAV-T20S-αΔN injected mice (Fig 5C) demonstrating that it is safe to express T20S-αΔN in rod photoreceptors by itself or with PAN^et^. However, expression of AAV-T20S-αΔN was weak and additional experiments are necessary to optimize T20S-αΔN for expression in photoreceptors. Future studies will test a more efficient IRES sequence and self-cleaving 2A peptides to improve coexpression of the T20S α and β- subunits. This was the first step toward augmenting protein degradation in mammalian neurons with archaeal proteasomes. Future experiments will evaluate whether T20S-αΔN improves vision in P23H and T17M rhodopsin mice and other neurodegenerative models [52] with protein-misfolding etiology.

## Conclusions

In summary, PAN^et^ provided a partial protective effect in P23H rhodopsin mice and expression of the gateless archaeal proteasome, T20S-αΔN is nontoxic for photoreceptors. Expression of archaeal proteasomes may be an effective therapeutic approach to increase resistance to proteotoxic stress in neurodegenerative diseases.

## Supporting information

S1 Fig 24 classes of T20-αΔN TEM images. (TIF)

S1 Raw images (PDF)

S1 File (CPPROJ)

S2 File (XLSX)

S3 File (XLSX)

S4 File (XLSX)

S5 File (XLSX)
