# Supplementary figures and images for: Therapeutic potential of archaeal unfoldase PANet and the gateless T20S proteasome in P23H rhodopsin retinitis pigmentosa mice
Source: PLoS One. 2024 Oct 3;19(10):e0308058. doi: 10.1371/journal.pone.0308058 (PMC11449290; doi:10.1371/journal.pone.0308058)

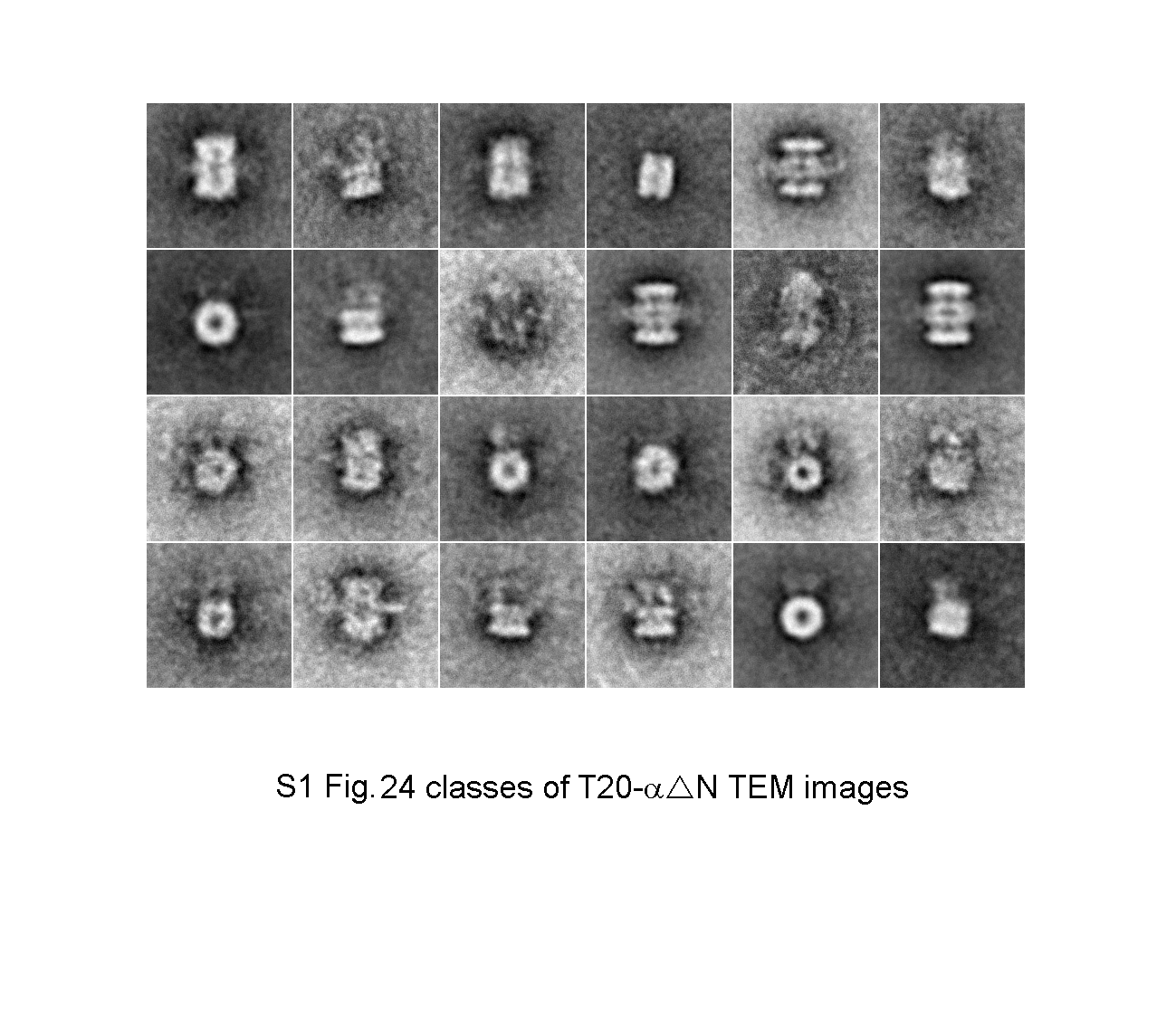

Supplement: S1 Fig — (TIF) [file pone.0308058.s001.tif]
