# Supplementary material for: Therapeutic potential of archaeal unfoldase PANet and the gateless T20S proteasome in P23H rhodopsin retinitis pigmentosa mice
Source: PLoS One. 2024 Oct 3;19(10):e0308058. doi: 10.1371/journal.pone.0308058 (PMC11449290; doi:10.1371/journal.pone.0308058)
Supplement: S1 Raw images — (PDF) [file pone.0308058.s002.pdf]

Fig 2A raw data
LiCore Odyssey
WB: anti-GFP/800

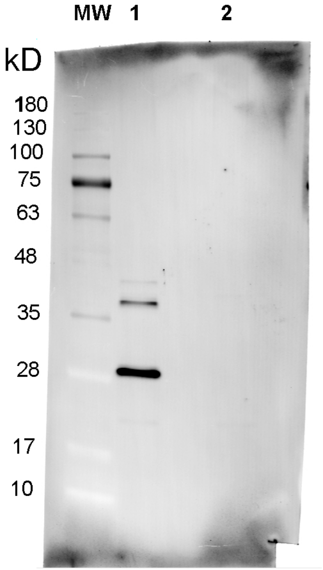

Gel: Novex10-20% Tris-Glycine

Fig 2A raw data
LiCore Odyssey
WB: anti-Ubiquitin/680

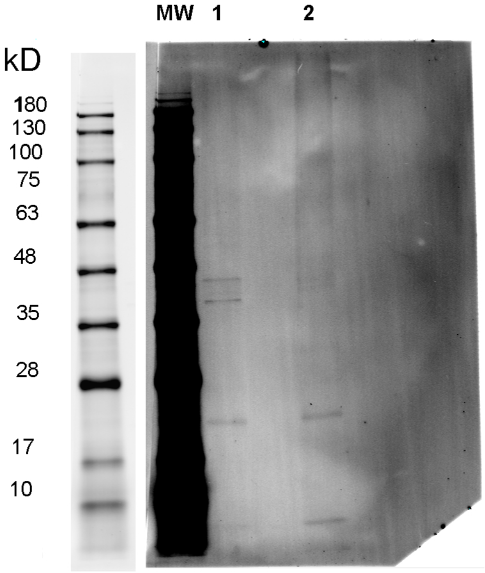

Gel: Novex10-20% Tris-Glycine

Fig 2B raw data
LiCore Odyssey
WB: anti-GFP/800

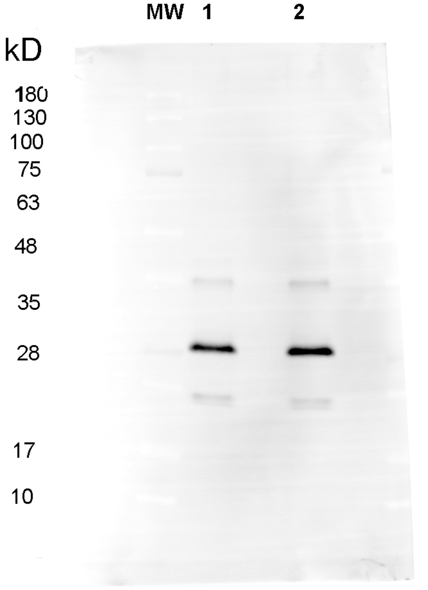

Gel: Novex10-20% Tris-Glycine

Fig 2B raw data
LiCore Odyssey
WB: anti-HA/680

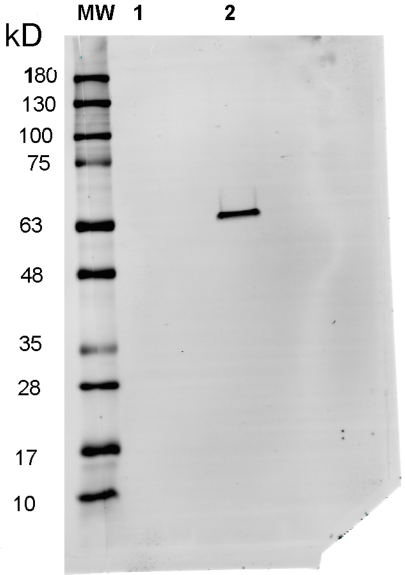

Gel: Novex10-20% Tris-Glycine

Fig 2B raw data
LiCore Odyssey
WB: anti-GFP/800

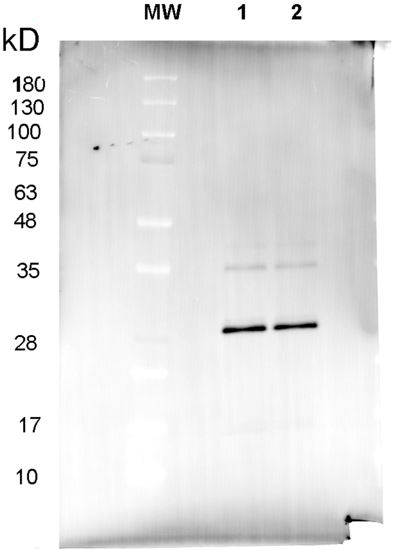

Gel: Novex10-20% Tris-Glycine

Fig 2B raw data
LiCore Odyssey
WB: anti-HA/680

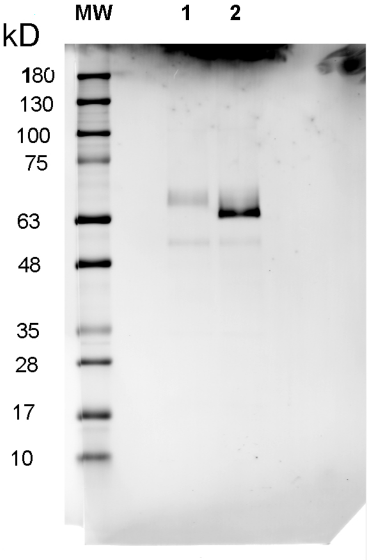

Gel: Novex10-20% Tris-Glycine

Fig 4C raw data
LiCore Odyssey
WB: anti-6x-His tag/680

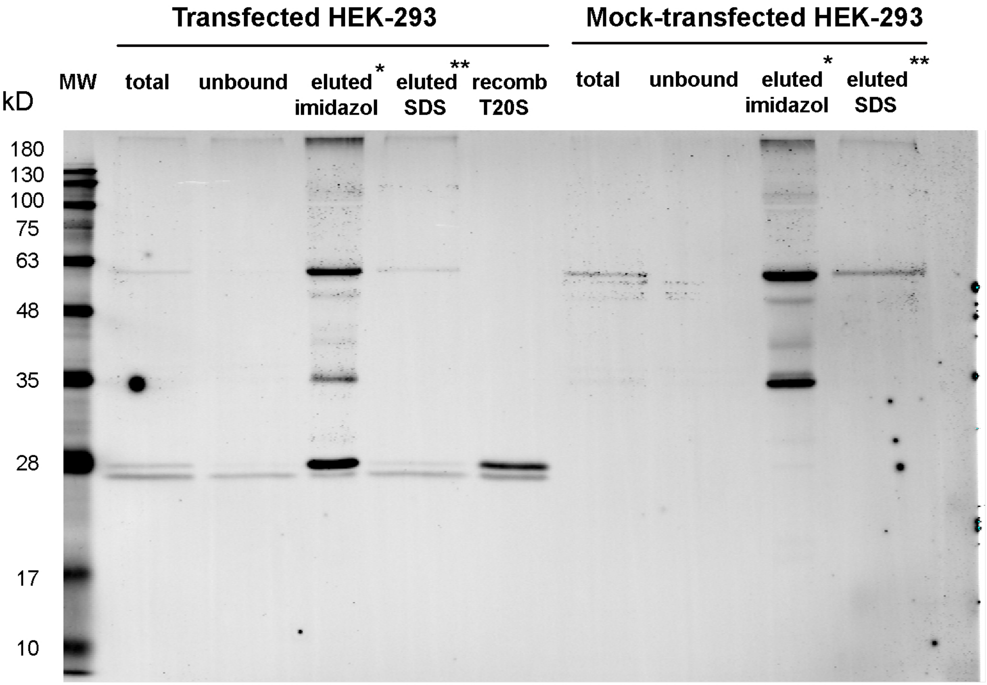

Gel: 12.5% Tris-Glycine

* Lane used in Fig 4C
** Additional elution with SDS after elution with inidazol

Fig 4C raw data
LiCore Odyssey
WB: anti-Proteasome 20S-α/680

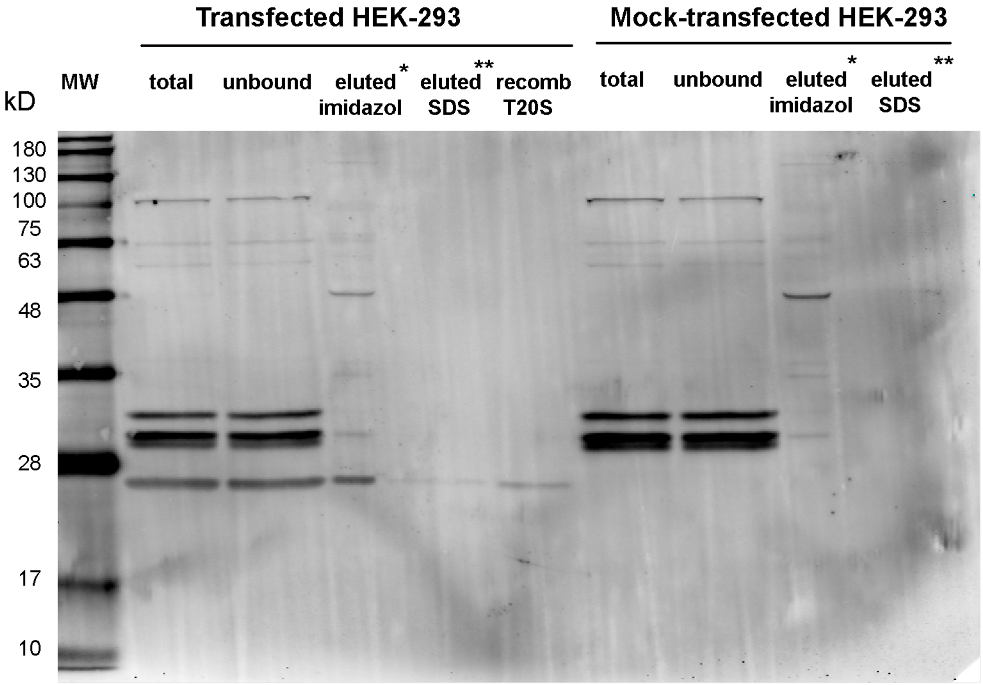

Gel: 12.5% Tris-Glycine

* Lane used in Fig 4C
** Additional elution with SDS after elution with inidazol

Fig 4C raw data
LiCore Odyssey
WB: anti-6x-His/680

anti-Proteasome 20S-α/680

MW 1 2 MW 1 2

180
130
100
75
63
48
35
28
17
10

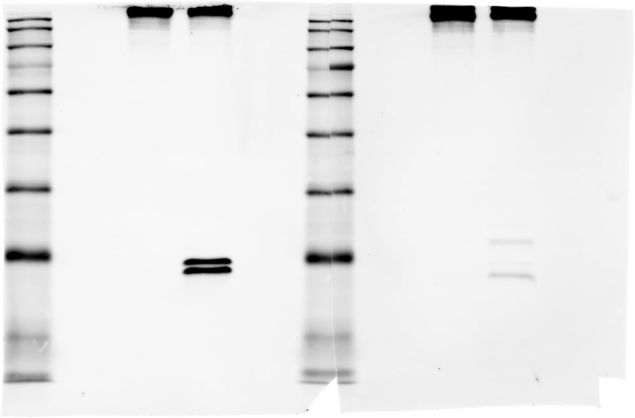

used in Fig 4

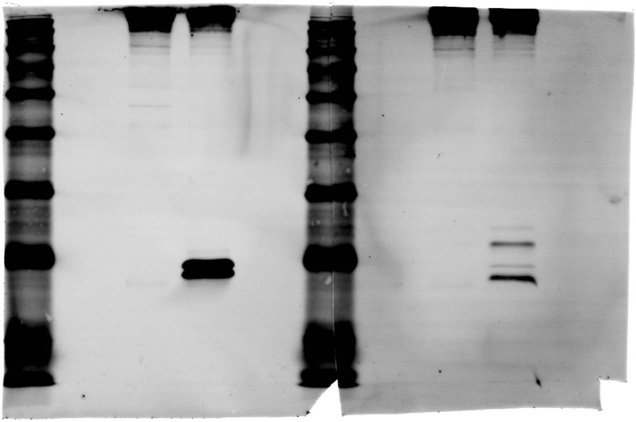

used in Fig 4

Gel: 12.5% Tris-Glycine

Fig 5B raw data

LiCore Odyssey

WB: anti-6x-His tag/680

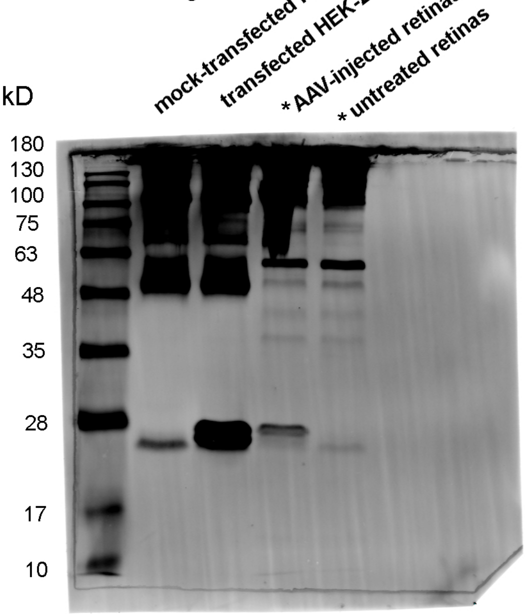

Gel: 12.5% Tris-Glycine

* Lane used in Fig 5B

Fig 5C raw data
LiCore Odyssey

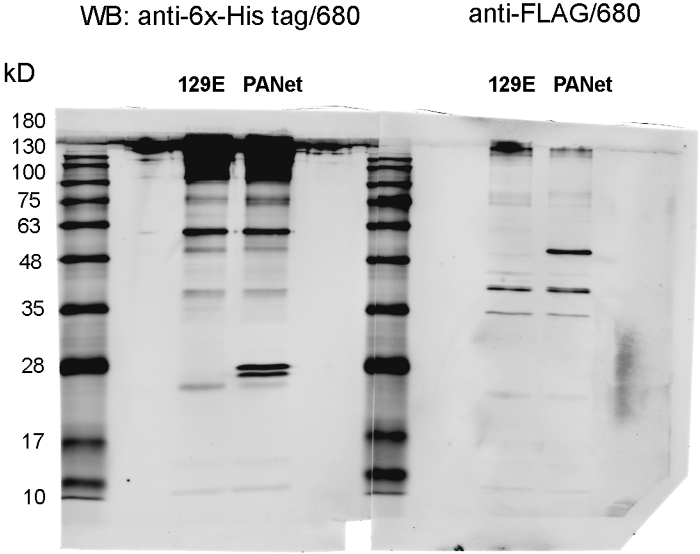

Gel: 12.5% Tris-Glycine
